# Slow Down and Concentrate: Time for a Paradigm Shift in Fall Prevention among People with Parkinson's Disease?

**DOI:** 10.1155/2013/704237

**Published:** 2013-02-24

**Authors:** Emma L. Stack, Helen C. Roberts

**Affiliations:** Academic Geriatric Medicine, Faculty of Medicine, University of Southampton, Mailpoint 807, University Hospital Southampton, Tremona Road, Southampton SO16 6YD, UK

## Abstract

*Introduction*. We know little about how environmental challenges beyond home exacerbate difficulty moving, leading to falls among people with Parkinson's (PwP). *Aims*. To survey falls beyond home, identifying challenges amenable to behaviour change. *Methods*. We distributed 380 questionnaires to PwP in Southern England, asking participants to count and describe falls beyond home in the previous 12 months. *Results*. Among 255 responses, 136 PwP (diagnosed a median 8 years) reported falling beyond home. They described 249 falls in detail, commonly falling forward after tripping in streets. *Single fallers* (one fall in 12 months) commonly missed their footing, walking, or changing position and recovered to standing alone or with unfamiliar help. *Repeat fallers* (median falls, two) commonly felt shaken or embarrassed and sought medical advice. *Very frequent fallers* (falling at least monthly; median falls beyond home, six) commonly fell backward, in shops and after collapse but often recovered to standing alone. *Conclusion*. Even independently active PwP who do not fall at home may fall beyond home, often after tripping. Falling beyond home may result in psychological and/or physical trauma (embarrassment if observed by strangers and/or injury if falling backwards onto a hard surface). Prevention requires vigilance and preparedness: slowing down and concentrating on a single task might effectively prevent falling.

## 1. Introduction

Postural instability is common in Parkinson's disease (PD): falls are likely to be a frequent problem for most people with Parkinson's (PwP) by 10 years after diagnosis [1]. Approximately one third of elderly people falls in any given year but approximately two thirds of PwP [2]. Most falls among PwP happen at home; for example, 80% of the 639 fall recorded over six months in one fall-prevention trial [3]. Current opinion about preventing falls in PD favours a multimodal approach: a combination of exercise and developing new movement strategies, coupled with optimal medical management [4].

PwP use attentional mechanisms and other cues to compensate for movement difficulty [5–7]: it is possible that they would find a distracting environment more challenging than one in which they could preserve focus. Small, qualitative studies have explored this possibility in relationship to walking, though not falls. As Lamont et al. wrote, after discussing community walking in focus groups with 18 PwP, “Challenging environments that demand attention may compromise the ability to walk” [8]. Jones et al. concluded from 20 semistructured interviews that “People with PD need to constantly assess and drive their walking performance” using “attentional resources, which can themselves be compromised” [9]. Most PwP experience progressive gait difficulties [10, 11]: it is possible that they would find unfamiliar environments progressively more challenging over time.

Yet we know little about the specific circumstances in which PwP fall beyond home. Li et al. described outdoor falls (three-quarters of which were “attributable to modifiable environmental factors”) as a “neglected public health problem” [12]. While previous studies on “community walking” [8, 9] have identified factors that help or hinder *walking*, they have not specifically identified factors that increased or decreased *stability*. If PwP learn to avoid and/or otherwise manage the key situations in which falls tend to happen, they may prevent a number of falls. 

We aimed to survey falls beyond home among PwP, identifying challenges that might be manageable through behaviour change. We proposed a postal survey to allow a wide cross-section of people to describe what had happened when they fell beyond their homes. 

## 2. Methods

We constructed a simple questionnaire addressing falls beyond home, that is, not in the familiar environment of their own home or garden but including allotments (small plots of publicly owned land rented to individuals, usually for growing vegetables) and the homes and gardens of friends and family. The questionnaire containing instructions throughout was accompanied with an information sheet and a stamped addressed reply envelope for postal return. We asked the participant's age and gender, date of diagnosis, use of mobility aids indoors and out, and how many times they had fallen at home and beyond in the previous 12 months. We did not define a fall in the questionnaire, offering respondents scope to describe what they considered a fall. We intended to disregard any descriptions in which the individual (a) did not come to rest on the ground or another lower level, (b) fell out of a bed or chair asleep, or (c) was knocked to the ground. We provided space for them to answer the following questions (used previously face-to-face and via falls diaries [13, 14]) for up to three of their most recent falls beyond home.

1. Where did you fall?
2. What were you doing?
3. Why do you think you fell?
4. How did you land?
5. Then what happened?

Limiting repeat fallers to describing no more than three falls meant that no individual or subgroup would dominate the snapshot of falls reported. Approximately 350 questionnaires were distributed via 30 branches of Parkinson's UK across Southern England. Additionally, via one geriatrician's clinic we handed out approximately 30 questionnaires to people who reported a fall beyond home during a consultation. Recipients took the questionnaires home to consider, complete, and/or post back. 

We entered all data received onto an excel spreadsheet, including all legible descriptions of falls. Using simple content analysis, we categorized and counted responses to each question. In reporting, we present totals for the whole sample, single fallers, and repeat fallers. To illustrate the circumstances in which very frequent fallers described falling, we present those who fell at least monthly separately from other repeat fallers (who fell from 2 to 11 times in 12 months). 

We conducted the study with the approval of the Southampton and South West Hampshire Research Ethics Committee (B).

## 3. Results

### 3.1. Participants

**Figure 1:**
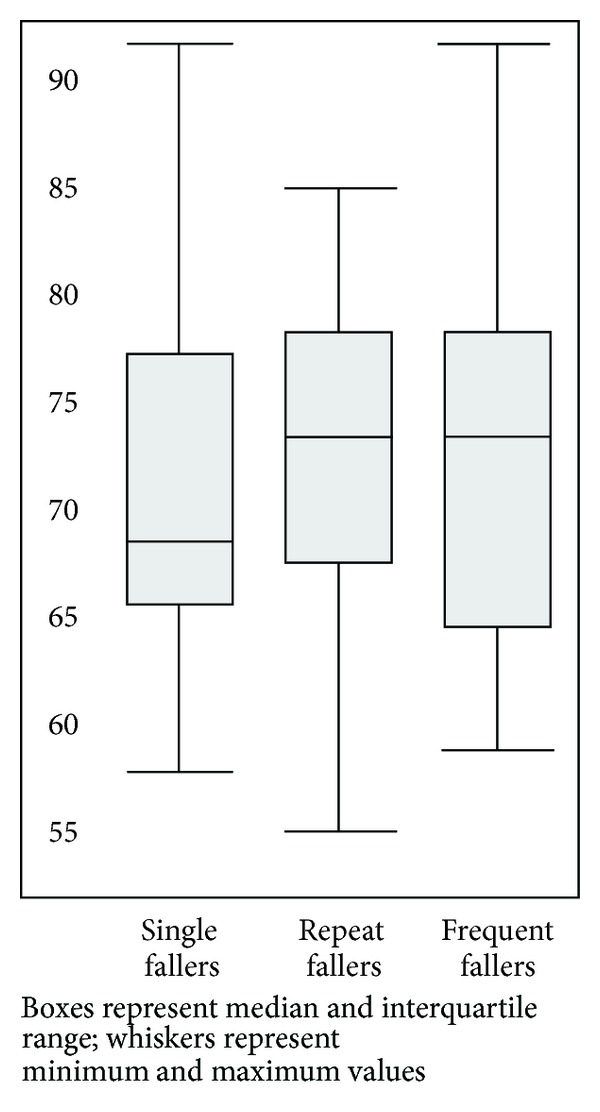
Box and Whisker Plot: Age (in years) by Group.

**Figure 2:**
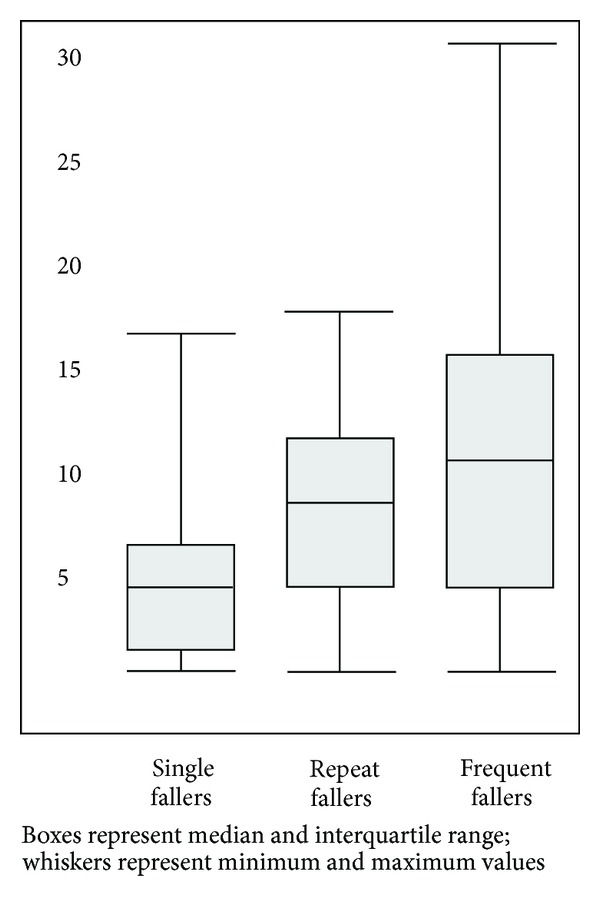
Box and whisker plot: time since diagnosis (in years) by group.

Of the 255 questionnaires returned, the 136 (53%) that contained descriptions of falls beyond home are included in the analysis presented here. In Table 1 and Figures 1 and 2, we summarise the characteristics of these 86 men and 50 women (aged from 54 to 91 years). We received responses from 19 single fallers, 86 repeat fallers (median 2 falls beyond home in a year), and 31 very frequent fallers (median 6 falls beyond home in a year).

### 3.2. Completeness of Data

Participants described the location of 249 falls in varying detail. In 240/249 descriptions (96%), we discerned the activity during which they fell, and, in 226 descriptions (91%), fallers proposed a cause. It was apparent how the faller landed in 216 descriptions (87%). Responses to “Then what happened?” often contained more than one type of answer, so we counted comments about recovery to standing (173 descriptions, 69%), injury (100, 40%), and immediate healthcare (40, 16%) separately, meaning that the total number of descriptions exceeded 249. Of the 100 comments about injury, 16 stated that the fall had not caused any injury; we thus collated 84 descriptions of physical or psychological injury. Some participants had a proxy write for them or typed a response. Three comments were illegible.

### 3.3. Circumstances of Falling

As shown in Table 2, 38% of falls occurred in streets or car parks and 35% in (or at the entrance of) unfamiliar buildings, commonly shops. The proportion of “green” falls was 13% in the countryside and 11% in unfamiliar gardens. 

Over half the falls (52%) occurred during walking. Other effortful (“strenuous”) activities, gardening, shopping, and playing with grandchildren, for example, accounted for 18% of falls. Attempting to enter or exit a vehicle (termed “vehicle transfers” in the table), predominantly cars, accounted for 9% of falls. Ascending and descending steps contributed equally to 6%. Falls from standing accounted for 5% of falls, as did standing from sitting or sitting from standing (sit or stand transfers in the table). 

Sudden causes of falling (like tripping) outweighed the failure to complete an action (like turning), see Table 3. Among sudden falls, tripping was most common, accounting for 24%. Inadequate concentration or vigilance, attributed to distraction or fatigue, accounted for a further 12%, followed in frequency by freezing (6%). Among the failures to complete an action, fallers referred to “loss of balance” in 18% of cases and turning in 9%. Causes grouped under “collapse” included “blood pressure,” “blacked out,” “legs buckled,” and “stimulator failed.”

Falling forward was most common, for example, on hands and knees (50%), with almost equal numbers of backward and sideways falls (18% each).

### 3.4. Immediate Consequences of Falls

The participants' reports of injury, recovery to standing and health service intervention following falls are summarised in Table 4. Participants mentioned minor injuries in 26% of descriptions and major injuries in 3%; they specified being uninjured in 6% of cases but feeling shaken or embarrassed in 5%. Respondents mentioned receiving health service intervention after approximately one in six falls beyond home (16%). In 30% of descriptions, fallers regained standing alone. In 20% of descriptions, someone familiar helped, most frequently their spouse, and, in 20%, fallers mentioned someone unknown, like a passerby, helping. Single fallers mostly recovered alone, but if they received help, it was mostly unfamiliar. Repeat- and very frequent fallers tended to receive help usually from someone they knew. 

### 3.5. Group Specific Features

The single faller group was of youngest median age and shortest median disease duration with similar numbers of men and women. The 1100% increase among those using a walking aid indoors and out was marked. This was the group most likely to report falling while walking or changing between sitting and standing, to mention minor (or no) injury and to report attending the Emergency Department. They were relatively likely to fall after tripping or missing their footing but relatively unlikely to attribute falling to distraction or fatigue. They were more likely than other groups to fall forwards (and less likely to fall sideways or backwards). They were relatively more likely to regain standing independently or with a stranger's help after falling (and less likely to receive help from someone familiar). 

The repeat fallers were the only participants to attribute falls to slipping, to report feeling shaken or embarrassed afterwards, to report seeing a doctor or nurse other than in the Emergency Department after falling, or to require hospital admission.

There were twice as many men as women among the very frequent fallers, who had the longest median disease duration and highest use of walking aids. They were more likely than other groups to fall backwards or sideways, in an unfamiliar building, after a collapse, and less likely to fall forwards, after tripping, in a green space. They were also relatively less likely to mention sustaining injury. 

## 4. Discussion

Response rate and data quality were satisfactory: we received replies to approximately two-thirds of the questionnaires distributed, from PwP diagnosed between 1 and 31 years, and had to disregard only three illegible answers. We had to look widely to capture 200-plus detailed descriptions of falls: while over half our respondents had fallen at least once beyond home, most falls occur at home. However few home environments pose the challenge of maintaining postural stability while attempting complex and/or strenuous movements, on changeable underfoot conditions, amidst much noise and congestion. 

Ashburn et al. reported that 80% of the falls they documented happened at home, in a study of similar sample size and participant age and disease duration as this study [3]. Prior to falling, their participants were ambulant (45%), standing (32%) and transferring (21%): they attributed 11% of falls to “freezing, festination and retropulsion.” In this study, we attributed a higher percentage of falls to “walking” and much smaller percentages to standing and transferring between sitting and standing. The differences in fall-related activities probably reflect differences in time use at and beyond home [13]. As we attributed only 6% of falls to freezing, it may be that starting to walk, passing through doorways, and turning round occupy proportionately less time outside the home than they do at home. There is evidence that abrupt movements frequently cause falls among PwP [14]: our respondents described falling trying to dodge people coming toward them or to step backward out of someone's way. PwP may be safer standing their ground and letting others dodge them.

Tripping after unwanted contact with the ground or other hazard dominated the causes of falling in this study and others [3, 15]. Streets are dense with trip hazards: doorways, kerbs, steps, street furniture, raised markings to guide people with visual impairments, and random, uneven paving slabs. Grass, sand, earth, and gravel contain natural variations and obscured obstacles (like tree roots) that similarly challenge ground clearance, particularly if an individual is fatigued or distracted. Ineffective foot clearance may reflect the “deteriorating and hypokinetic motor control” underlying many falls [16]. 

Standing up from the ground, likely to be necessary after falling outdoors (in the absence of seating), challenges strength, flexibility, and balance. Fewer respondents described injuries than mentioned needing help to stand up. We observed potentially important differences in the proportion of our subgroups falling backward or sideways, which may follow the loss of intersegmental flexibility (or “stiffness”) [17]. Falling in any direction other than forwards exposes the greater trochanter to direct trauma on landing with a high risk of significant injury [18, 19]. Interestingly, in the current study, only 10/78 (13%) of the very frequent fallers' descriptions of falling mentioned sustaining any physical injury, despite nearly half being described as backward or sideways. It may be that limiting respondents to describing a maximum three falls deterred them from reporting their most serious falls or that those who fall very frequently “learn” to land safely. Both suggestions require further research. 

Using an appropriate walking aid outdoors may afford extra postural stability and prevent falls: (only one single faller used an aid indoors, while 12 took one outside), but devices blocking forward motion and occupying both hands may contemporaneously increase the chance of a backward landing if stability is lost. We found the single fallers least likely to fall backward or sideways and the very frequent fallers the least likely to fall forwards, a difference possibly attributable to greater stiffness among the latter or to their greater use of mobility aids. 

Besides the injuries sustained when falling backward, these falls may be particularly embarrassing. Falling into the unknown, sustaining injury, and/or being publically embarrassed can leave the faller “shaken” or otherwise distressed. Repeat fallers were the only group to report being “shaken” by falling. Occasional falls in “embarrassing” circumstances (backwards in public and needing help to get up) may be more emotionally disturbing than frequent falls of which PwP may have learned to expect and manage or a single fall from which they are quickly back on their feet. The “shock” of a rare fall may lead some individuals (not just frequent fallers) to exhibit a fear of falling [20]. Those without a history of falls are more likely to leave home unaccompanied and are perhaps more likely to find strangers offering help if they should fall, like the single fallers in the current study. Teaching PwP how best to direct a “helper” after a fall could be as useful as teaching them how to get up alone. 

PwP use their attention (and other cues) to generate and maintain movement. They seem most at risk of falls when they are distracted and fail to focus on movement. One similarity between our findings and those of Ashburn et al. [3] is in the 12% of falls they attributed to “misjudgement and distraction” and the 15% we attributed to “distraction” and “missed footing.” Even among the single fallers in the current study, tripping, distraction, and missed footing accounted for 12 of the 13 falls with a sudden cause (92%). No strategy will be universally applicable, but our finding that over 40% of the falls reported were attributed to tripping, distraction, missed footing, or rushing suggests that hypervigilance may be an effective and widely applicable fall-prevention strategy for PwP. For PwP and significant cognitive impairment, hypervigilance may be a strategy more effectively employed by an accompanying carer.

Some argue that exercise aimed at increasing muscle strength, postural stability, and joint flexibility, coupled with learning new strategies for tackling challenging movements (like turns and transfers), is an effective way of reducing falls among PwP [21]. An exercise-based intervention is not universally applicable (nor desirable) [22, 23], and the supporting evidence is not strong [24]. In a recent review and meta-analysis, falls have not been among the list of outcomes for which physiotherapy was found to benefit people with Parkinson's [24]. A recent trial in which 67 PwP exercised at home for six weeks and 67 served as controls has demonstrated no significant difference in the rates of falling at eight weeks or six months though near misses reduced significantly among those who exercised [21]. Li et al. stated that “no clinical trial has shown the efficacy of exercise in reducing falls” among PwP but went on to generate evidence for a reduction in falls after Tai Chi [25]. We agree that there is some evidence to support both exercise and the learning of new strategies (e.g., dual-task training) to improve mobility: “fall rates may underestimate positive effects of exercise” [26] if they are not adjusted for activity level. However, it is worth considering also training PwP to focus on one activity at a time, given the difficulties that PwP experience when multitasking. Learning to focus on mobility and avoid distractions surely deserves a place among the growing raft of strategies we advocate for safe mobility. PwP will need to take care to avoid trips, survive freezing episodes, and turn safely (for example) no matter how fit they are. 

Despite considerable research, improved medical management of PD, and heightened awareness of the potential benefits of exercise, fall frequency among PwP shows little evidence of decreasing. We are not the first to question whether we have “oversold the benefit of late-life exercise” and to suggest that while acknowledging the current balance of evidence, we need to look beyond exercise to more “behavioural” strategies that may benefit individuals [27]. A simple change in behaviour, slowing down to focus on mobility rather than any external distractions (e.g., chatting or rushing), may effectively prevent falls and may also be faster, cheaper, and more widely applicable than some exercise-based interventions.

Effective exercise “contributes to both fitness and fatigue” [28]. Exercise-induced fatigue can impede postural control even in healthy young subjects [29]: even local muscle fatigue in the ankle dorsiflexors may diminish ground clearance. Among PwP, high background muscle tone may exacerbate physical fatigue, and the need to concentrate for long periods, applying attention to once “automatic” movements, may exacerbate mental fatigue. Thus, anyone using hypervigilance to the environment (and his or her mobility therein) as a fall avoidance strategy must couple it with a sensitivity to fatigue. If they do not rest when fatigued, physically or mentally, people may make errors of judgement [30], which could lead to falls. 

We call this change from the promotion of exercise and environmental modification toward hypervigilance and fatigue management a paradigm shift because it necessitates a change in the basic assumptions made and held by the scientific community [31]. Instead of focusing on negatives, like a lack of movement speed or postural stability, we advocate focusing on excess (muscle tone, mental demand, anxiety, or fatigue, e.g.). “Slowing down and taking care” is at least as valid fall-prevention strategy as exercise and warrants further research. This study supports recent findings about PwP walking in the community, including their use of “consciously attending to walking” and “planning and preparation” as facilitating strategies and the significance of the external environment's demands as barriers [8]. 

This study allowed over 200 people to contribute to the study, including those with restricted mobility. Our respondents may have exhibited volunteer bias, as most were able to attend branch meetings, and being members of a support network may distinguish them from other PwP: however, data saturation was reached. The results, based on cross-sectional data supplied by respondents, could be subject to recall bias. However, in this exploratory study we did not demand total recall: we wished to know how PwP would describe their experiences. While respondents in any falls study may forget certain details, we are unaware of any evidence to suggest that they fabricate events. We have commented both on what respondents reported and where data was incomplete, which further allows the reader to assess the validity of the data. Furthermore, the subgroup analysis prevented falls by very frequent fallers dominating the picture. Future longitudinal research would illuminate the changing circumstances of falling, as some active single fallers progress to very frequent fallers over time. 

## 5. Conclusion

Even independently active PwP who do not fall at home do fall in the environment beyond home (often repeatedly). Unseen or unsuccessfully negotiated physical hazards (like uneven pavements and tight parking spaces) provide frequent challenges, compounded by psychological pressures (like multitasking or rushing).

Falls alter in type as they become more frequent. Hard, backward landings, which are difficult to control and from which it is difficult to recover without help replace trips in the street, from which the uninjured faller can recover to their feet independently: rescue, assessment, and treatment by the health services become more likely. Anyone falling alone and outside is likely to be observed and assisted by someone unfamiliar. A stranger's intervention in a public fall may heighten the immediate embarrassment and have disabling consequences. 

Avoiding falls in the environment beyond home requires vigilance and preparedness. Slowing down and concentrating on a single task (without unnecessary distraction) might reduce falls more effectively than waiting for the effects of an exercise programme to afford some protection and/or for pavements to be better maintained: testing this hypothesis warrants further research. 

## Figures and Tables

**Table 1:** Characteristics of participants.

		All	Group		
			Single fallers	Repeat fallers	Very frequent fallers
Participants	*N* (%)	136	19 (14)	86 (63)	31 (23)
Falls	*N* (%)	249	19 (8)	152 (61)	78 (31)
Gender	M : F	86 : 50	10 : 9	55 : 31	21 : 10
Age in years	Median (IQR)	72 (66–77)	68 (65–77)	73 (67–78)	73 (64–78)
	Min–Max	54–91	57–91	54–85	58–91
Years diagnosed	Median (IQR)	8 (4–12)	5 (2–7)	9 (5–12)	11 (5–16)
	Min–Max	1–31	1–17	1–18	1–31
Falls at home	Median (IQR)	2 (0–6)	0	1 (0–3)	18 (10–33)
Falls beyond home	Median (IQR)	2 (1–3)	1 (1-1)	2 (1–3)	6 (3–12)
	Min–Max	1–60	1-1	1–11	1–60
Walking aid indoors	*N* (%)	49 (36)	1 (5)	27 (31)	21 (68)
Walking aid outdoors	*N* (%)	86 (63)	12 (63)	49 (57)	25 (81)
Change in to out	% increase	76	1100	81	19
Falls described/faller	Median	2	1	2	3

*N*: number; %: percent; M : F: male to females; IQR: interquartile range.

**Table 2:** Falls beyond home: locations and fall-related activity (*N* = 249).

		All	Group		
			Single fallers	Repeat fallers	Very frequent fallers
Falls (*N*)		249	19	152	78

Location	Street/car park	95 (38%)	8 (42%)	58 (38%)	29 (37%)
	Buildings	85 (34%)	6 (32%)	47 (31%)	32 (41%)
	Green spaces	59 (24%)	5 (26%)	39 (26%)	15 (19%)
	In transit	10 (4%)	0	8 (5%)	2 (3%)
	Total	**249**	**19**	**152**	**78**

Activity	Walking	129 (52%)	11 (58%)	79 (52%)	39 (50%)
	Strenuous (including shopping)	44 (18%)	4 (21%)	28 (18%)	12 (15%)
	Vehicle transfers	22 (9%)	1 (5%)	13 (9%)	8 (10%)
	On steps	16 (6%)	1 (5%)	12 (8%)	4 (5%)
	Standing	12 (5%)	0	7 (5%)	5 (6%)
	Sit or stand transfers	12 (5%)	2 (11%)	5 (3%)	5 (6%)
	Toileting/dressing	5 (2%)	0	3 (2%)	2 (3%)
	Total	**240 (96%)**	**19 (100%)**	**146 (96%)**	**75 (96%)**

*N*: number, %: percent.

**Table 3:** Falls beyond home: causes and landings (*N* = 249).

		All	Group		
			Single fallers	Repeat fallers	Very frequent fallers
Falls (*N*)		249	19	152	78

Cause: sudden	Tripped	60 (24%)	9 (47%)	37 (24%)	14 (18%)
	Distracted or tired	29 (12%)	1 (5%)	17 (11%)	11 (14%)
	Freezing	16 (6%)	0	10 (7%)	6 (8%)
	Slipped	10 (4%)	0	10 (7%)	0
	Missed footing	9 (4%)	2 (11%)	6 (4%)	1 (1%)
	“Collapse”	9 (4%)	1 (5%)	1 (1%)	7 (9%)
	Total	**133 (53%)**	**13 (68%)**	**81 (53%)**	**39 (50%)**

Cause: failure	Lost balance	45 (18%)	3 (16%)	30 (20%)	12 (15%)
	Turned (including too fast)	23 (9%)	2 (11%)	14 (9%)	7 (9%)
	Rushing	10 (4%)	0	7 (5%)	3 (4%)
	Dodging someone	7 (3%)	0	4 (3%)	3 (4%)
	Reaching or bending	5 (2%)	0	4 (3%)	1 (1%)
	Step back/small space	3 (1%)	0	1 (1%)	2 (3%)
	Total	**93 (37%)**	**5 (26%)**	**60 (39%)**	**28 (36%)**

Landing	Forward	125 (50%)	14 (74%)	81 (53%)	30 (38%)
	Backwards/sideways	91 (37%)	3 (16%)	54 (36%)	34 (44%)
	Total	**216 (87%)**	**17 (89%)**	**135 (89%)**	**64 (82%)**

*N*: number, %: percent.

**Table 4:** Immediate consequences of falls beyond home (*N* = 249).

		All	Group		
			Single	Repeat	Very frequent
Falls (*N*)		249	19	152	78

Injury	Minor injuries	45 (18%)	5 (26%)	32 (21%)	8 (10%)
	Head or facial injury	19 (8%)	2 (11%)	16 (11%)	1 (1%)
	No injury*	16 (6%)	2 (11%)	8 (5%)	6 (8%)
	Shaken or embarrassed	12 (5%)	0	12 (8%)	0
	Fracture/dislocation	8 (3%)	1 (5%)	6 (4%)	1 (1%)
	Total	**100 (40%)**	**10 (53%)**	**74 (49%)**	**16 (21%)**

Recovery	Stood alone	75 (30%)	7 (37%)	40 (26%)	28 (36%)
	Stood with known help	49 (20%)	1 (5%)	30 (20%)	18 (23%)
	Stood with stranger's help	49 (20%)	5 (26%)	27 (18%)	17 (22%)
	Total	**173 (69%)**	**13 (68%)**	**97 (64%)**	**63 (81%)**

Input	Paramedics attended	15 (6%)	1 (5%)	9 (6%)	5 (6%)
	Attended A and E	16 (6%)	2 (11%)	13 (9%)	1 (1%)
	Seen by doctor or nurse	7 (3%)	0	7 (5%)	0
	Admitted to hospital	2 (1%)	0	2 (1%)	0
	Total	**40 (16%)**	**3 (16%)**	**31 (20%)**	**6 (8%)**

*N*: number, %: percent, *signifies that the comment was “no injury sustained,” for example.
